# Supplementary material for: Natural hybridization in heliconiine butterflies: the species boundary as a continuum
Source: BMC Evol Biol. 2007 Feb 23;7:28. doi: 10.1186/1471-2148-7-28 (PMC1821009; doi:10.1186/1471-2148-7-28)
Supplement: Additional File 1 — Hybrids between species of Heliconius and Eueides butterflies: a database. HTML file linking to database of all known wild-caught interspecific hybrid specimens in the Heliconiina, consisting of introductory text, a list of specimens, together with collection data and photographs of the specimens, and links to information about some artificial hybrids and mutants in the group. This is an edited copy of our online database of Heliconius hybrids [102]. To view database, download zip file and extract to a separate folder, then open index.html within that folder. [file 1471-2148-7-28-S1.zip › hecele01.html]

hybrid hecele01


---


Hybrid between *H. hecale* (or possibly *Heliconius ?numata*)
and *H. elevatus zoelleri*
Colombia
© J.F. Le Crom

Return to table
of hybrids

To next hybrid
  
To previous hybrid

```
NOTES

No:                      14
Genus of species 1:      Heliconius
Species 1:               hecale?
Subspecies of species 1: ?
Genus of species 2:      Heliconius
Species 2:               elevatus
Subspecies of species 2: zoelleri
Sex:                     f
Country:                 Colombia
Locality:                Guainia: Puerto In�rida
Year:                    1974
Photo no.:               hecele01
Named hybrid:
Collection:              Schmidt-Mumm/Instituto Alexander von Humboldt
Collector:               E. Schmidt-Mumm
Author/publication:      Velez & Salazar 1991: p. 132
Notes:                   BC->elevatus? on basis of phenotype; hecale as parent on basis of scalloped hindwing margin
```

**Last updated:** 30 July 2006

---
